# Design, Synthesis, and Biological Evaluation of Novel Tomentosin Derivatives in NMDA-Induced Excitotoxicity

**DOI:** 10.3390/ph15040421

**Published:** 2022-03-30

**Authors:** Mohamed Zaki, Mohammed Loubidi, Tuğçe Bilgiç, Derviş Birim, Mohamed Akssira, Taner Dagcı, Sabine Berteina-Raboin, Luciano Saso, Mostafa Khouili, Güliz Armagan

**Affiliations:** 1 Institut de Chimie Organique et Analytique ICOA, Pôle de Chimie, Université d’Orléans, UMR CNRS 7311, Rue de Chartres-BP 6759, CEDEX 2, 45067 Orléans, France; zaki.smc@gmail.com (M.Z.); m.loubidi@gmail.com (M.L.); 2 Department of Science, Ecole Normale Supérieure, Moulay Ismail University, BP. 3104, Toulal, Meknés 50000, Morocco; 3 Laboratoire de Chimie Physique & Biotechnologie des Biomolécules et des Matériaux, Université Hassan II Casablanca, FST, BP 146, Mohammedia 28800, Morocco; akssira.m@gmail.com; 4 Department of Physiology, Faculty of Medicine, Ege University, Bornova, 35100 Izmir, Turkey; tugce_bilgic@icloud.com (T.B.); taner.dagci@ege.edu.tr (T.D.); 5 Department of Biochemistry, Faculty of Pharmacy, Ege University, Bornova, 35100 Izmir, Turkey; dervis.birim@ege.edu.tr; 6 Department of Physiology and Pharmacology Vittorio Erspamer, Sapienza University of Rome, P. le Aldo Moro 5, 00185 Rome, Italy; luciano.saso@uniroma1.it; 7 Laboratoire de Chimie Moléculaire, Matériaux et Catalyse, Faculté des Sciences et Techniques, Université Sultan Moulay Slimane, Campus Mghilla, BP 523, Beni-Mellal 23000, Morocco; M.KHOUILI@usms.ma

**Keywords:** oxidative stress, excitotoxicity, apoptosis, amino-tomentosin, 1,2,3-triazolo-tomentosin

## Abstract

N-methyl-D-aspartate (NMDA) receptor stimulation may lead to excitotoxicity, which triggers neuronal death in brain disorders. In addition to current clinical therapeutic approaches, treatment strategies by phytochemicals or their derivatives are under investigation for neurodegenerative diseases. In the present study, novel amino and 1,2,3-triazole derivatives of tomentosin were prepared and tested for their protective and anti-apoptotic effects in NMDA-induced excitotoxicity. Amino-tomentosin derivatives were generated through a diastereoselective conjugate addition of several secondary amines to the α-methylene-γ-butyrolactone function, while the 1,2,3-triazolo-tomentosin was prepared by a regioselective Michael-type addition carried out in the presence of trimethylsilyl azide (TMSN_3_) and the α-methylene-γ-lactone function. The intermediate key thus obtained underwent 1,3-dipolar Huisgen cycloaddition using a wide range of terminal alkynes. The possible effects of the derivatives on cell viability and free-radical production following NMDA treatment were measured by Water-Soluble Tetrazolium Salts (WST-1) and Dichlorofluorescein Diacetate (DCF-DA) assays, respectively. The alterations in apoptosis-related proteins were examined by Western blot technique. Our study provides evidence that synthesized triazolo- and amino-tomentosin derivatives show neuroprotective effects by increasing cellular viability, decreasing ROS production, and increasing the Bcl-2/Bax ratio in NMDA-induced excitotoxicity. The findings highlight particularly **2e**, **2g**, and **6d** as potential regulators and neuroprotective agents in NMDA overactivation.

## 1. Introduction

Neurodegenerative disorders [1] are progressive diseases that are characterized by the death of specific nerve cells [2]. N-methyl-D-aspartate (NMDA) receptors are widely expressed in the central nervous system [3]. The overactivation of NMDA receptors causes a higher intracellular Ca^+2^ influx, which is the major cause of neuron injury with some apoptotic pathways [4,5]. Apoptosis is a programmed cell death that requires self-destructive, active RNA/protein synthesis and energy. Mitochondria are the most important organelles in apoptosis and are regulated by members of the Bcl-2 family. The Bcl-2 family includes both pro-apoptotic (Bax, Bad, Bak, Bim, Bcl-Xs, Bid) and anti-apoptotic (Bcl-2, Mcl-1) members [6,7,8]. Apoptotic cell death induced by excitotoxicity has been demonstrated in many experimental models [9,10,11,12].

An allosteric conformational change promotes the oligomerization of Bax and Bak proteins in the outer mitochondrial membrane. Oligomerization of these proapoptotic proteins produces pores in the mitochondria resulting in the loss of cytochrome c [13]. The X protein (Bax) associated with Bcl-2 supports apoptosis. Bax-defective neurons and Bax-deficient animals were found resistant to apoptosis. The synergistic effect of their deletion provokes an overall increase in cell count in many areas of the brain [14].

In an attempt to address the previous issue, several studies have been conducted on sesquiterpene lactones because they have various biological properties and are a rich source of drug candidates in different stages of clinical trials [15,16,17]. The biological properties of sesquiterpene lactones are partly due to the existence of the α-methylene-γ-lactone moiety as a potential alkylating agent. Either as extracts or essential oil, Dittrichia viscosa L. Greuter, an invasive and perennial plant, is exploited in traditional Moroccan herbal medicine as an anti-inflammatory, antipyretic, and antiseptic agent [18,19]. This perennial flowering plant is an abundant source of lactones, in particular tomentosin 1, also called xanthalongin, that is readily extracted in 1.5% of its total dry weight from the aerial part of the plant [20,21,22]. Tomentosin has attracted increasing interest from researchers due to its inexpensive production and broad spectrum of biological activities as a cytotoxic and anti-inflammatory compound [23,24,25,26]. We report herein an evaluation of the protective and anti-apoptotic effects of amino and 1,2,3-triazole derivatives of tomentosin in NMDA-induced excitotoxicity.

## 2. Results and Discussion

### 2.1. Chemistry

**Scheme 1:**
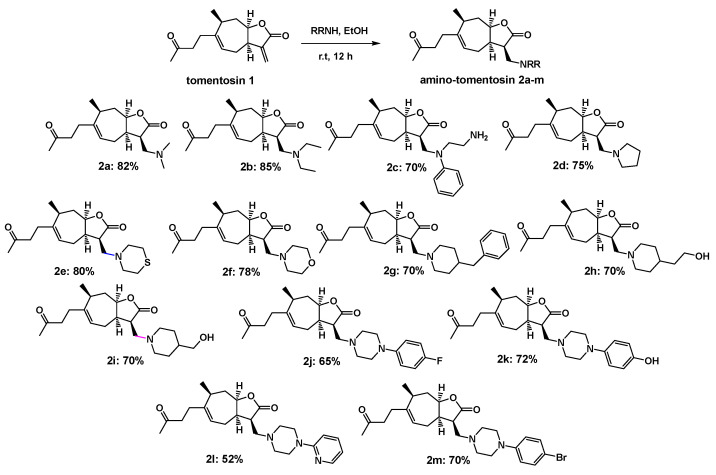
Synthesis of various amino-tomentosins.

The amino-lactone derivatives of tomentosin were efficiently synthesized by a Michael addition reaction of various secondary amines to the α-methylene-γ-butyrolactone function of tomentosin **1**. The desired products **2a**–**m** were synthesized in good yields (Scheme 1) [27].

**Scheme 2:**
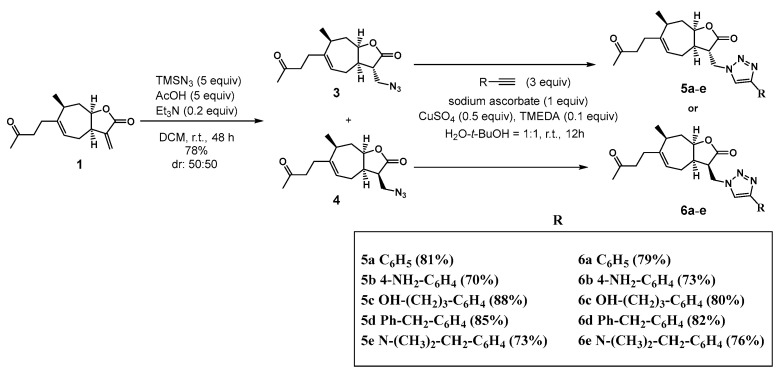
Synthesis of 1,2,3-triazole-tomentosins.

In order to synthetize a series of 1,2,3-triazole tomentosin scaffolds, trimethylsilyl azide (TMSN_3_) and α-methylene-γ-lactone **1** from natural sesquiterpene were engaged in a Michael-type addition reaction. The reaction led to products **3** and **4** in a diastereomeric mixture which was easily separated by column chromatography. These compounds were then reacted under Huisgen 1,3-dipolar cycloaddition reaction conditions by using different terminal alkynes. The compounds **5a**–**e** and **6a**–**e** obtained are depicted in Scheme 2 [28].

### 2.2. Biological Studies

#### 2.2.1. Cell Viability Significantly Increased following Treatment with Tomentosin Derivatives

Inhibition of NMDAR-mediated excitotoxicity is among the powerful therapeutic strategies for brain disorders. Plant-derived compounds such as ibogaine and huperzine A have been shown to inhibit NMDA and glutamate-induced toxicity [29,30]. Although they have high affinity and selectivity, these drugs that act on a single target may not be effective for the treatment of the disease. In our study model, the protective effects of amino- and triazolo-tomentosin derivatives were assessed in an excitotoxicity model in which toxicity was triggered by 2 mM NMDA by means of cellular viability, ROS production, and regulation in proapoptotic and antiapoptotic protein levels. The alteration in cell viability was examined following treatments to determine the possible regulation by the synthesized compounds. Previously, Yang et al. [31] reported that tomentosin (IC50 = 10 µM) showed anticancer activity in cancer cells. Thus, all derivatives, alone, at 10 µM were exposed to cells for 24 h before NMDA studies. None of them reduced cell viability when compared to untreated cells. Cell viability was found between 94.56% and 102.81% in these groups. Following the confirmation of nontoxicity of derivatives, experiments with NMDA-treated cells were performed. The cells were pre-treated with the synthesized compounds (0.01 to 10 μM) for 2 h, followed by 2 mM NMDA exposure for an additional 1 h. The WST-1 assay was used to quantify cell proliferation and viability. Results showed that NMDA-induced cellular death was decreased by the synthesized compounds (Table 1 and Table 2). The cell viability detected in NMDA-treated cells was 52.14% ± 3.36. The amino derivatives **2c**, **2d** at 0.1 µM then **2e**, **2g**, **2b** at 1 µM, and the triazolo derivatives **5a**, **6a**, **5d**, and **6d** at 0.1 µM, provided higher than 50% cellular protection against NMDAR-mediated excitotoxicity.

#### 2.2.2. Tomentosin Derivatives Prepared to Decrease Free-Radical Production

**Figure 1:**
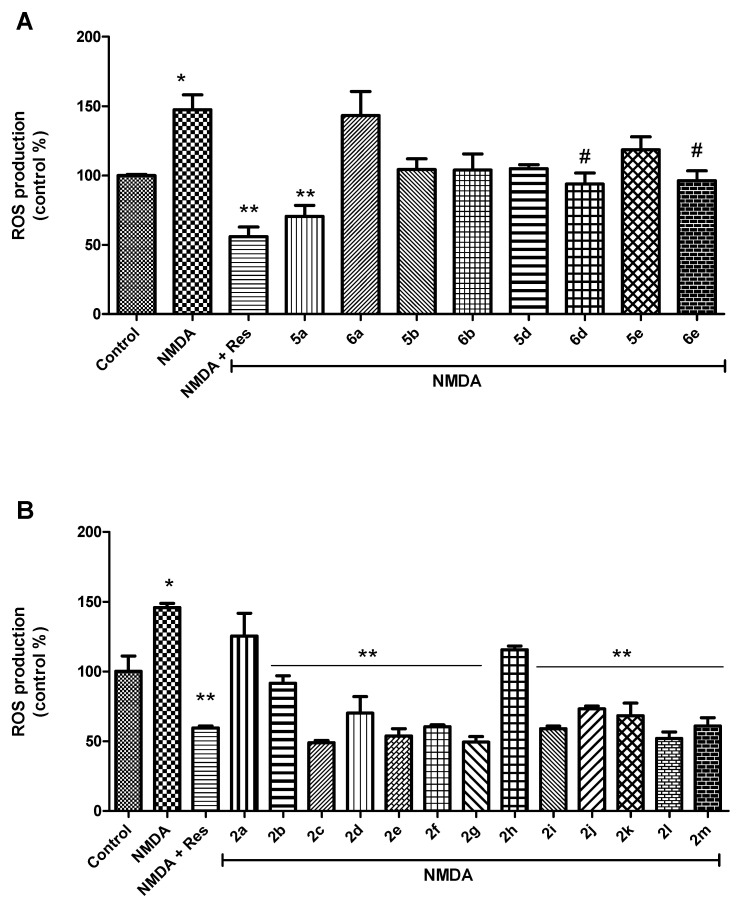
The alteration in ROS production following (**A**) triazolo- and (**B**) amino-tomentosin treatments at indicated concentrations in NMDA (2 mM)-treated SH-SY5Y cells. The concentration of each derivative was 0.1 µM, except for **2b**, **2e**, and **2g**, whose concentrations were 1 µM. * *p* < 0.05 significant difference from untreated cells; ** *p* < 0.01 significant difference from NMDA-treated cells; # *p* < 0.05 significant difference from NMDA-treated cells.

The final in-cell response of many neurotoxic stimuli is oxidative stress, which is thought to trigger apoptosis in many acute and chronic neurodegenerative diseases [32,33]. Oxidative stress-related pathological alterations including the accumulation of ROS, damage to macromolecules, and a significant reduction in endogenous antioxidants are observed in the pathophysiology of neurodegeneration. Consequently, plant-derived natural compounds that exhibit antioxidant properties can contribute significantly to the treatment of neurodegenerative diseases. In our previous studies, we showed that NMDAR-mediated cell death is accompanied by increased ROS production and macromolecular damage [34,35,36]. In this study, we found that the high ROS production following NMDA treatment is further evidence of the induction of oxidative stress in neurons (Figure 1). To evaluate the possible role of tomentosin derivatives on oxidative stress in NMDA receptor activated-cells, a DCF-DA assay was used to measure cellular ROS. Resveratrol was used as a well-known antioxidant to compare the efficiency of synthesized derivatives. As shown in Figure 1, an increase in the fluorescence intensity of DCF was detected after NMDA exposure or NMDA and derivative treatments. These results show that receptor overactivation triggers free-radical generation in cells. However, incubation with the triazolo-derivatives **5a**, **6d**, and **6e**, in the presence of NMDA (70.45 ± 13.65, 93.84 ± 13.66, 96.18 ± 12.46%, respectively), significantly decreased ROS production in cells when compared with NMDA-treated cells (147.48 ± 18.34%, respectively) (*p* < 0.05, Figure 1A). At the indicated concentration (0.1 µM), cell viability was determined 79, 77, and 67% following **5a, 6d**, and **6e** treatments, respectively, as shown in Table 1, consistent with a higher ROS-scavenging activity. Similarly, most of the amino-tomentosin derivatives at indicated concentrations succeeded in reducing the fluorescence intensity of DCF in the presence of NMDA (*p* < 0.01, Figure 1B). These consistent results between cellular protection and ROS reduction following treatments may provide opportunities to improve the studies on tomentosin derivatives for the management of disorders influenced by free radicals.

#### 2.2.3. Bcl-2/Bax Ratio Was Altered by Tomentosin Derivatives

**Figure 2:**
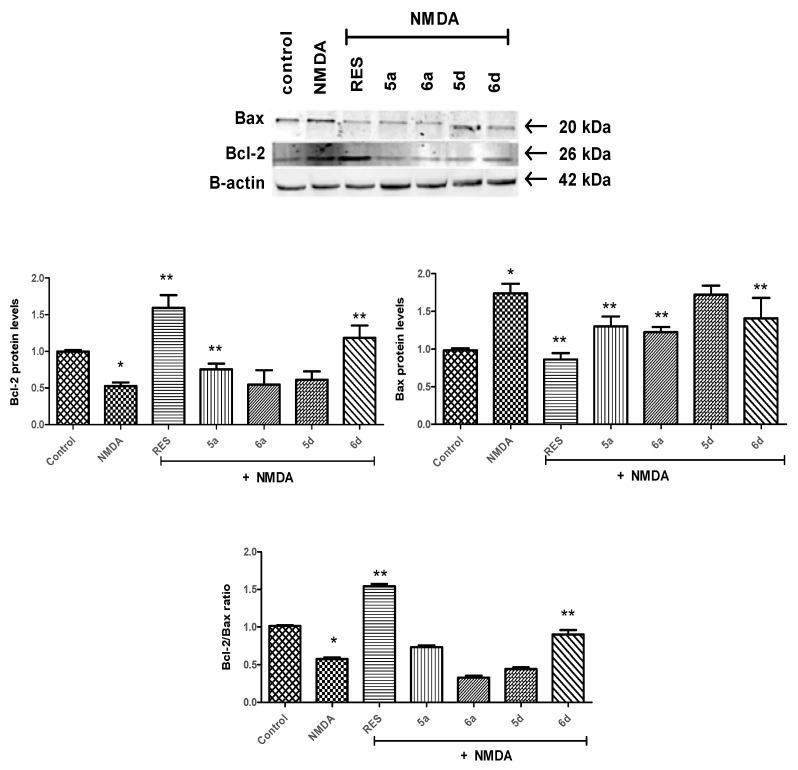
The alteration in Bax and Bcl-2 protein levels following 0.1 µM triazolo-tomentosin derivative treatments in NMDA-treated cells. Quantified band values were normalized to the corresponding β-actin signal. Bar graph data represent the mean ± SD; *n* = 3 independent experiments. * *p* < 0.01 significant difference from untreated cells; ** *p* < 0.001 significant difference from NMDA-treated cells.

**Figure 3:**
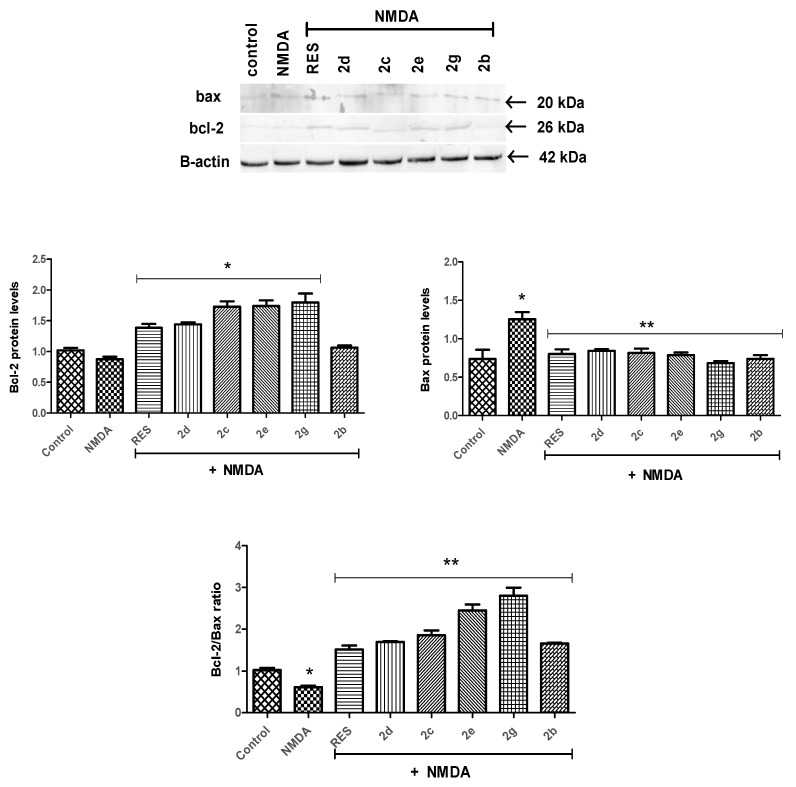
The alteration in Bax and Bcl-2 protein levels following amino-tomentosin derivative treatments in NMDA-treated cells. The concentration of **2c** and **2d** was 0.1 µM and the concentration of **2b**, **2e**, and **2g** was 1 µM. Quantified band values were normalized to the corresponding β-actin signal. Bar graph data represent the mean ± SD; *n* = 3 independent experiments. * *p* < 0.05 significant difference from untreated cells; ** *p* < 0.01 significant difference from NMDA-treated cells.

In previous studies, tomentosin was shown to decrease cytokine release and, similar to other sesquiterpene lactones, the presence of the acetyl moieties in tomentosin is suggested to be related to its anti-inflammatory activity. In our study, the levels of apoptosis-related proteins were examined to evaluate the anti-apoptotic potential of the synthesized compounds in NMDAR-mediated excitotoxicity. Among the results obtained by cell viability analysis, the compounds showing more than 50% protection in the presence of NMDA were chosen to determine the possible involvement in apoptosis. Figure 2 and Figure 3 show that NMDA-treated cells had elevated Bax levels and reduced Bcl-2 levels compared to untreated cells (*p* < 0.05). Regarding the triazolo-tomentosin derivatives, although Bax levels were significantly reduced by **5a**, **6a**, and **6d** treatments, similar changes supporting their anti-apoptotic roles were only observed when cells were treated by **6d**. When compared to triazolo-tomentosin derivatives (Figure 2), all selected amino-tomentosin derivatives (except **2b**) were found to decrease Bax and increase Bcl-2 protein levels (Figure 3). The Bcl-2: Bax ratio is crucial for the inhibition or induction of cellular death and is known to have a greater influence than the individual expression of each protein in determining susceptibility to apoptotic stimuli in cells. Therefore, the ratio between Bcl-2 and Bax was determined following the treatments in our study. The elevated Bax/Bcl-2 expression ratio produced by NMDA was remarkably lowered by **6d** and all selected amino-tomentosin derivatives. Particularly, inhibition of cell death by **2e**, **2g**, and **6d** with increasing Bcl-2/Bax ratio is promising for neurodegeneration studies. These observations supported the hypothesis that tomentosin derivatives could provide a neuroprotective effect through regulating protein expressions which are responsible for mitochondrial membrane pore formation.

## 3. Materials and Methods

### 3.1. Chemistry Methods

#### 3.1.1. Synthesis of Amino-Tomentosin **2a**–**m**

To a solution of tomentosin 1 (1 eq) in ethanol (10 mL), a secondary amine (1.1 eq) was added and the mixture was stirred for 12 h at room temperature. Ethanol was evaporated, then water (10 mL) and AcOEt (10 mL) were added and the resulting aqueous phase was extracted with AcOEt (3 × 10 mL). The organic phases were dried over MgSO_4_, filtered, and concentrated under reduced pressure. The amino-tomentosins were obtained with good yields after purification by flash chromatography on silica gel.

#### 3.1.2. Synthesis of 13-Azido-11,13-Dihydrotomentosin **3** and **4**

The mixture of trimethylsilyl azide (TMSN_3_) (5 eq) and AcOH (5 eq) in 15 mL of anhydrous CH_2_Cl_2_ was stirred for 20 min at room temperature. Then, tomentosin 1 (1 eq) and a catalytic amount of triethylamine (0.8 eq) were introduced under an argon atmosphere and the reaction was left at room temperature for 48 h. Water (10 mL) was next added and the aqueous phase was extracted with AcOEt (3 × 10 mL). The combined organic phases were washed with a saturated solution of sodium bicarbonate (3 × 10 mL), brine (3 × 10 mL), and dried over MgSO_4_, filtered, and concentrated under reduced pressure. The products **3** and **4** were obtained after purification in 78% global yield (1:1).

#### 3.1.3. Synthesis of 1,2,3-Triazolo-Tomentosin **5a**–**e** and **6a**–**e**

To a product **3** or **4** in tert-butyl alcohol-water (1:1, 10 mL) was added sodium ascorbate (1 equiv.), copper sulfate (0.5 equiv.), tetramethylenediamine (0.1 equiv.), and the appropriate alkyne (3 equiv.). Then, the mixture was stirred at room temperature for 12 h under an argon atmosphere. Water (5 mL) was added and the aqueous phase was extracted (3 × 10 mL) with AcOEt. The combined organic phase was washed with brine, dried over MgSO_4_, filtered, and concentrated under reduced pressure. The desired product was obtained after purification by flash chromatography on silica gel (petroleum ether–AcOEt).

## 4. Biological Methods and Materials

### 4.1. Materials

Dulbecco’s Modified Eagle’s Medium (DMEM) and fetal bovine serum (FBS) were purchased from Life Technologies. Primary antibodies of Bax (#2774) and Bcl-2 (#15071) were obtained from Cell Signaling Technology. N-methyl-D-aspartate (NMDA) and 5-(and-6)-chloromethyl-2′,7′-dichlorodihydrofluorescein diacetate (H2DCF-DA) were obtained from Sigma Aldrich. Penicillin/streptomycin was purchased from Invitrogen.

### 4.2. Cell Culture and Treatments

The human neuroblastoma cell line (SH-SY5Y) was obtained from the American Type Culture Collection (ATCC, Catalog #CRL-2266). Cells were suspended in DMEM containing 10% FBS and 1% penicillin and streptomycin (100 U/mL). Following seeding, cells were differentiated with 10 μM all-trans-retinoic acid (RA) for 7 days in medium containing FBS 1%. WST-1 and DCF-DA assays were performed in 96-well plates (2 × 103 cells/well). For WST-1, the cells were pre-treated with compounds (0.01–10 µM) in fresh medium and incubated for 2 h. Then, in the presence of compounds, cells were treated with 2 mM NMDA for additional 1 h. Following the incubations, the culture medium was then replaced with fresh medium and incubated for 12 h. Protein analyses were performed in six-well plates (5 × 105 cells/well). At first, all the tested compounds were prepared as 5 mM in sterile dimethylsulfoxide (DMSO). The DMSO final working concentration was less than 0.1%.

### 4.3. Cell Viability Assay

Cell viability was assessed using the WST-1 assay. Ten microliters of WST-1 was added to wells following exposure and the plates were incubated for 3 h. The absorbance was measured at 570 nm and 630 nm using a microplate reader (VersaMax, Molecular Devices LLC, San Jose CA, USA). All results were normalized to 100% using the vehicle control cells.

### 4.4. Free-Radical Generation (DCF-DA Assay)

ROS was measured by the fluorescence intensity of DCF, with an excitation wavelength of 485 nm and an emission wavelength of 535 nm as described previously [37,38]. All results were normalized to 100% using the vehicle control cells.

### 4.5. Protein Analysis

The synthesized compounds at indicated concentrations (amino derivatives **2c**, **2d** at 0.1 µM; **2b**, **2e**, **2g** at 1 µM; triazolo derivatives **5a**, **6a**, **5d**, and **6d** at 1 µM) were exposed to SH-SY5Y cells (5 × 105 cell/well) for 2 h. After 2 h, the cells were treated with 2 mM NMDA for 1 h. Following the incubations, the culture medium was then replaced with fresh medium and incubated at 37 °C in a 5% CO_2_ incubator for 12 h. Then, the cells were washed with PBS and lysed in 1X radioimmunoprecipitation assay (RIPA) buffer containing protease inhibitor cocktail. Following centrifugation at 10,000× *g* for 10 min at 4 °C, supernatant was taken to determine total protein concentration by the Bicinchoninic acid (BCA) protein assay. Western blot assays were performed by using 30 µg of protein and densitometric analyses of bands were performed as described previously [38].

### 4.6. Statistical Analysis

Data are presented as means ± standard deviation (SD). The statistical significance was evaluated using one-way ANOVA followed by Tukey’s post hoc tests. Values of *p* < 0.05 were considered statistically significant.

## 5. Conclusions

Natural compounds that exhibit pharmacological properties act as important guides when synthesizing novel multi-targeted molecules. Although tomentosin has been shown to induce apoptosis in cancer cells [39,40,41], its cell-protective potential should be taken into consideration [26]. In this study, novel synthesized triazolo- and amino-derivatives at selected concentrations were observed to reverse cellular death and change pro-/anti-apoptotic protein levels. The α-methylene-γ-lactone nucleus plays an important role in biological effects including cytotoxic, anti-inflammatory, and neuroprotective actions. It is seemed that specific structural moieties of sesquiterpene lactone alter its activity and that a significant increase in Bcl-2/Bax ratio could be the molecular mechanism behind derivative-mediated protection against NMDA-induced cell death. The current study establishes the potential of tomentosin derivatives, particularly **2e**, **2g**, and **6d**, as neuroprotective agents. Taken together, tomentosin derivatives-induced neuroprotection was partially due to regulation of neuronal apoptosis in excitotoxic conditions. In addition, these compounds may directly or indirectly influence oxidative stress, which is involved in several neurodegenerative diseases.

## Figures and Tables

**Table 1:** Cell viability results following treatment with amino-tomentosin derivatives against NMDAR-mediated excitotoxicity. All values are means ± SDs (*n* = 5) and *p* < 0.05 significant difference from NMDA-treated cells.

	% Cell Viability			
Compound	10 µM	1 µM	0.1 µM	0.01 µM
**2a**	57.95 ± 0.96	66.74 ± 2.06	71.78 ± 8.02	59.14 ± 4.78
**2b**	70.58 ± 5.96	77.37 ± 9.29 ^#^	72.45 ± 7.83	67.16 ± 6.15
**2c**	68.95 ± 3.17	70.42 ± 3.57	82.89 ± 12.01 ^#^	65.49 ± 1.63
**2d**	72.11 ± 4.76	69.74 ± 4.46	105.8 ± 4.70 ^#^	101.15 ± 8.55 ^#^
**2e**	67.32 ± 1.51	80.74 ± 4.07 ^#^	63.78 ± 2.35	67.16 ± 6.81
**2f**	70.00 ± 2.63	63.74 ± 3.23	71.45 ± 3.25	64.42 ± 2.11
**2g**	75.74 ± 10.84	83.63 ± 12.47 ^#^	70.62 ± 3.23	64.56 ± 4.23
**2h**	61.32 ± 1.27	66.11 ± 4.36	71.51 ± 3.57	73.59 ± 9.11
**2i**	67.21 ± 0.92	73.11 ± 4.37	70.90 ± 5.56	65.16 ± 3.22
**2j**	68.63 ± 2.01	74.79 ± 10.73	74.69 ± 5.44	59.47 ± 4.23
**2k**	71.21 ± 4.45	67.42 ± 6.72	67.37 ± 7.03	62.75 ± 1.94
**2l**	64.21 ± 2.94	64.63 ± 2.69	63.17 ± 3.66	63.22 ± 3.28
**2m**	69.37 ± 2.24	64.16 ± 2.39	68.18 ± 7.18	63.42 ± 6.82
**2 mM NMDA**	52.14 ± 3.36 **			

** *p* < 0.05 significant difference from control cells. # Neuroprotection at the indicated concentration was found to be higher than 50%. Values represent the mean ± SDs of five independent determinations.

**Table 2:** Cell viability results following treatment with triazolo-tomentosin derivatives against NMDAR-mediated excitotoxicity, *p* < 0.05 significant difference from NMDA-treated cells.

	% Cell Viability			
Compound	10 µM	1 µM	0.1 µM	0.01 µM
**5a**	61.11 ± 0.97	63.83 ± 0.56	79.44 ± 1.51 ^#^	65.02 ± 3.49
**6a**	58.00 ± 1.35	60.20 ± 1.78	77.50 ± 3.42 ^#^	62.42 ± 3.23
**5b**	58.82 ± 1.88	59.73 ± 3.16	65.56 ± 4.81	61.95 ± 2.61
**6b**	58.78 ± 3.49	60.11 ± 0.66	66.83 ± 2.09	62.01 ± 3.12
**5c**	56.40 ± 1.88	61.24 ± 4.63	65.76 ± 2.45	60.68 ± 2.65
**6c**	55.67 ± 1.95	57.91 ± 3.56	66.23 ± 3.09	62.15 ± 0.92
**5d**	57.96 ± 3.45	58.52 ± 0.81	77.53 ± 2.71 ^#^	60.34 ± 1.11
**6d**	55.93 ± 1.68	56.01 ± 2.91	77.90 ± 3.88 ^#^	60.21 ± 1.93
**5e**	57.14 ± 1.10	56.66 ± 2.91	63.95 ± 2.25	60.81 ± 0.87
**6e**	60.37 ± 0.95	59.73 ± 1.17	67.37 ± 2.73	63.49 ± 1.22
**2 mM NMDA**	52.14 ± 3.36 **			

** *p* < 0.05 significant difference from control cells. # Neuroprotection at the indicated concentration was found to be higher than 50%. Values represent the mean ± SDs of five independent determinations.

## Data Availability

Data is contained within the article.
